# Endoscopic repair of lateral sphenoid Encephaloceles: a case series

**DOI:** 10.1186/s12901-017-0044-x

**Published:** 2017-11-28

**Authors:** Mitchell R. Gore

**Affiliations:** Department of Otolaryngology, SUNY-Upstate Medical University, Physicians Office Building North, Suite 4P 4900 Broad Road Syracuse, Syracuse, NY 13215 USA

**Keywords:** Cerebrospinal fluid leak, Lateral sphenoid encephalocele, Nasoseptal flap

## Abstract

**Background:**

Lateral sphenoid encephaloceles present a surgical challenge. These encephaloceles may be difficult to access given their lateral location and proximity to the neural and vascular structures of the sphenoid floor, pterygopalatine fossa, and lateral and superior sphenoid walls. Additionally, many patients have idiopathic intracranial hypertension, increasing the risk of recurrence. When untreated or undiscovered, these encephaloceles increase the risk of meningitis.

**Methods:**

All consecutive endoscopic repairs of lateral sphenoid encephaloceles by a single surgeon from 2012 to 2017 were analyzed for method of repair, complications, and recurrence rate. Odds ratio for recurrence of CSF leak for Alloderm inlay/abdominal fat sphenoid obliteration/nasoseptal flap with multilayer repair vs. other method (Alloderm onlay/contralateral nasoseptal flap or free mucosal graft) was compared, and Fischer’s exact test was used to calculate the two-sided *p*-value for the two repair methods.

**Results:**

The success rate (no recurrence of cerebrospinal fluid rhinorrhea) for Alloderm inlay/abdominal fat onlay/nasoseptal flap onlay was 100% while for Alloderm onlay/contralateral nasoseptal flap + free mucosal graft the success rate was 0%. For any nasoseptal flap repair vs. free mucosal graft the success rates were 83.3% and 16.7% respectively. The success rate for Alloderm inlay/abdominal fat onlay/nasoseptal flap onlay vs. Alloderm onlay/contralateral nasoseptal flap + free mucosal graft was statistically significant (*p* = 0.048), but the success rate for any nasoseptal flap repair vs. free mucosal graft was not significant (*p* = 0.29). The success rate for patients without post-op lumbar drain vs. with post-op lumbar drain was also nonsignificant (*p* = 0.29).

**Conclusions:**

In the author’s hands Alloderm inlay/abdominal fat onlay/nasoseptal flap onlay was superior to other repair methods (Alloderm onlay/contralateral nasoseptal flap or free middle turbinate mucosa onlay graft). The complication rate was low. Post-operative lumbar drainage did not affect the success rate.

## Background

Lateral sphenoid sinus meningoceles, meningoencephloceles, and encophaloceles are relatively uncommon anatomic phenomena. These are often chronically developing abnormalities that may be related to chronically elevated intracranial/cerebrospinal fluid pressure (such as that seen in idiopathic intracranial hypertension) causing gradual erosion of the sphenoid roof and allowing herniation of the inferior temporal lobe into the lateral sphenoid recess. The lateral sphenoid recess, if pneumatized, is bounded by the foramen rotundum superiorly, internal carotid posteriorly, pterygopalatine fossa anteriorly, and Vidian canal inferiorly, making access to and safe dissection in this area challenging. Additionally, the frequent chronically elevated CSF pressure and three-dimensional anatomy at this site may make durable, “water-tight” repair a surgical challenge. Several recent case series [1, 2] have explored the use of endoscopic transsphenoidal repair, with a transpterygoid extension often necessary. Success rates are high with multilayered repair techniques and an endoscopic approach.

In this case series, the author presents a consecutive series of patients with lateral sphenoid encephaloceles who underwent endoscopic repair, to analyze the relationship between repair method and success rate, as well as to explore repair techniques, utility of post-operative lumbar drainage, and complication rates.

## Methods

**Fig. 1:**
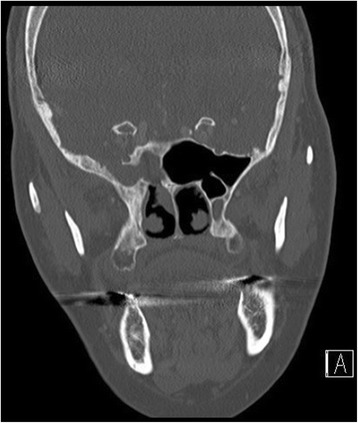
Coronal CT showing right lateral sphenoid defect and encephalocele in patient #5

**Fig. 2:**
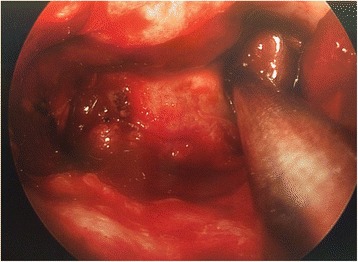
Exposed right lateral sphenoid defect in patient #5

**Fig. 3:**
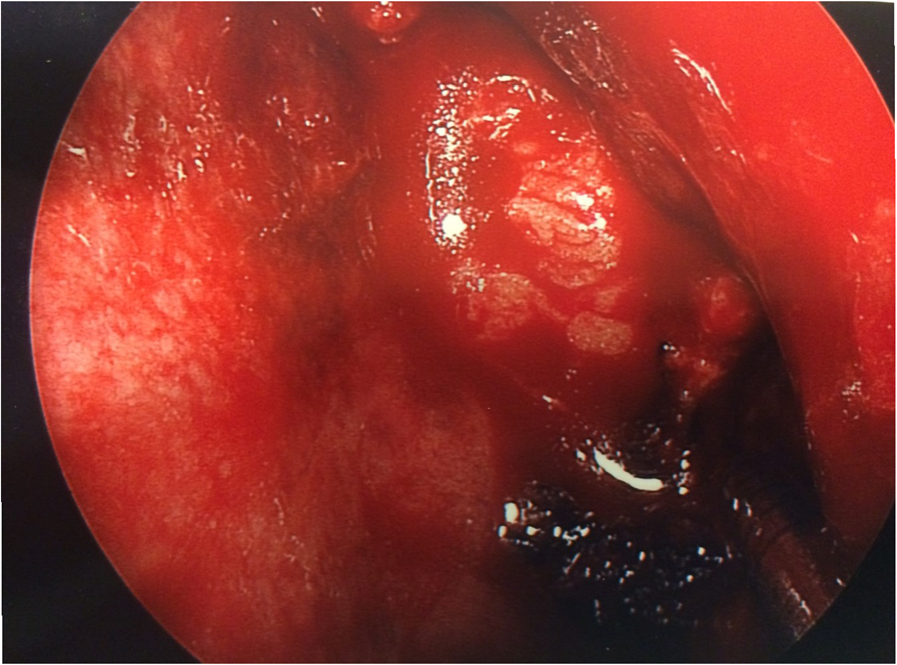
Abdominal fat onlay graft in left sphenoid in patient #4

**Fig. 4:**
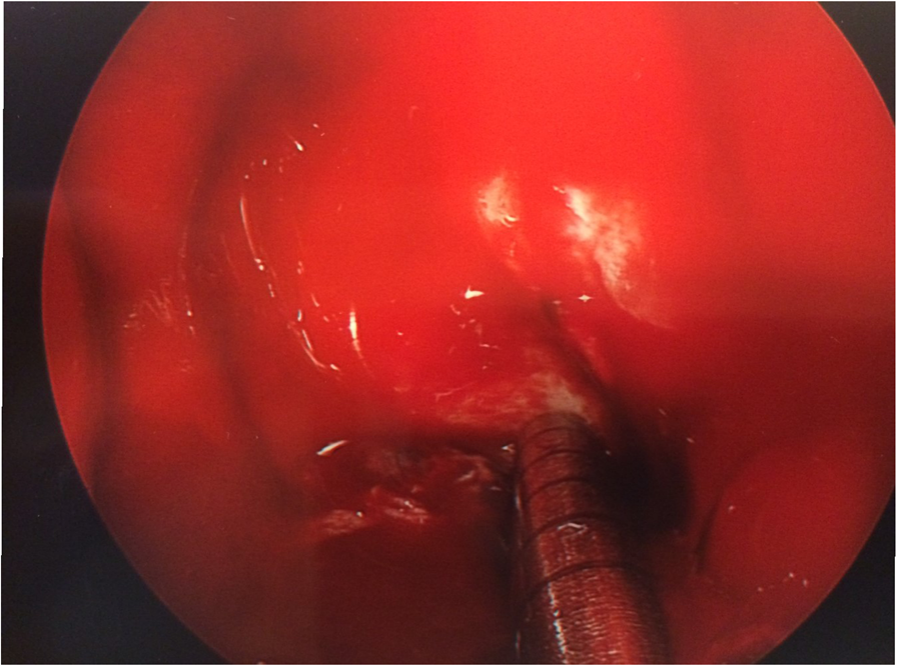
Ipsilateral left nasoseptal flap in place in left sphenoid in patient #4

The aim of this study was to analyze a series of seven patients who underwent endoscopic repair of lateral sphenoid encephaloceles diagnosed and treated between 2012 and 2017, and to examine the relationship between success rate and method of surgical repair. Figure 1 shows a coronal CT image illustrating a right lateral sphenoid encephalocele in patient #5. All patients were operated on by a single author at a tertiary care regional hospital. All patients underwent transnasal transsphenoidal endoscopic repair of a lateral sphenoid encephalocele. In all cases the lateral sphenoid encephalocele was widely exposed using an extended transsphenoidal approach (with removal of the posterior maxillary sinus wall and reflection of the pterygopalatine fossa contents inferiorly when necessary for exposure) and then reduced/excised using bipolar cautery until the defect was flush with the dura. Figure 2 shows the right lateral sphenoid encephalocele in patient #5 after wide sphenoidotomy and bipolar cauterization of the lateral sphenoid encephlocele. During exposure of the lateral sphenoid encephalocele the rescue flap modification of the nasoseptal flap was used to preserve the nasoseptal flap pedicle when an ipsilateral nasoseptal flap was utilized [3–5]. The nasoseptal flap was otherwise elevated and inlaid in the standard fashion [6]. Patient 1 underwent repair using an Alloderm (Lifecell/Kinetic Concepts, Inc., San Antonio, Texas) inlay/abdominal fat onlay/contralateral nasoseptal flap. Patient 2 underwent repair using Alloderm onlay/contralateral nasoseptal flap. Patient 3 underwent repair with an ipsilateral middle turbinate free mucosal onlay graft. Patients 4–7 underwent repair with Alloderm inlay/abdominal fat onlay/ipsilateral nasoseptal flap. Figure 3 shows the abdominal fat onlay graft in place in patient #4, while Fig. 4 shows the ipsilateral nasoseptal flap in place overlying the abdominal fat graft in patient #4. Patients were instructed to avoid lifting >25 pounds for 6 weeks post-operatively and all repairs were bolstered with Surgicel (Ethicon, Inc., Somerville, NJ), Duraseal Sealant (Integra Lifesciences Corporation, Plainsboro, NJ), and Nasopore (Stryker, Kalamazoo, Michigan). Patients 1 and 3 had lumbar drains placed intraoperatively by a Neurosurgeon and were drained at 10 ml per hour for 48 h, clamped for 24 h, and then had the drains removed after 72 h total. Odds ratio analysis was performed using MedCalc software (MedCalc Software, Inc., Ostend, Belgium) and two-sided *p* value/number needed to treat was determined using Fischer’s exact test using GraphPad Software (GraphPad Software, Inc., La Jolla, CA). *P*-value less than 0.05 was considered statistically significant. This study was determined to be exempt by the SUNY-Upstate Medical University Institutional Review Board.

## Results

The patient demographics, encephalocele location, repair method, outcome, and complications are noted in Table 1. Patients 2 (Alloderm onlay and contralateral nasoseptal flap) and patient 3 (ipsilateral middle turbinate free mucosal graft onlay) experienced recurrent CSF leaks as evidenced by recurrence of CSF rhinorrhea (patient 2) and recurrence of CSF rhinorrhea as well as evidence of recurrent lateral sphenoid encephalocele on subsequent MRI ordered for headaches (patient 3). Patient 2 was referred to another skull base surgeon and underwent revision of the nasoseptal flap, with resolution of the CSF rhinorrhea after the second procedure, while patient 3 was lost to follow up. Patients 1 (Alloderm inlay/abdominal fat onlay/contralateral nasoseptal flap) and patients 4–7 (Alloderm inlay/abdominal fat onlay/ipsilateral nasoseptal flap) all had resolution of their CSF rhinorrhea and no subsequent recurrence at the time of this study. The risk of recurrence for Alloderm inlay/abdominal fat onlay/nasoseptal flap (patients 1 and 4–7) was compared to the risk of recurrence for the combined other two methods (Alloderm onlay and contralateral nasoseptal flap in patient 2 and ipsilateral middle turbinate mucosal graft onlay in patient 3) using Fischer’s exact test. This was found to be statistically significant, with the two-sided *p* = 0.048, with a number needed to treat (NNT) of 1. Fischer’s exact test was also used to compare the risk of CSF leak recurrence with any nasoseptal flap (patients 1, 2, and 4–7) to free mucosal graft only (patient 3). This was nonsignificant, with *p* = 0.29. Similarly, when patients treated with post-op lumbar drainage (patients 1 and 3) were compared to patients who did not have lumbar drainage post-operatively (patients 2 and 4–7), no statistical difference was found for risk of recurrence (p = 0.29). The complication rate was low, and other than the recurrent CSF rhinorrhea noted in patients 2 and 3, the only other complication noted was right (ipsilateral to the encephalocele) dry eye in patient 5. She was examined by Ophthalmology at 8 weeks post-op and the tear production and visual acuity/eye exam were noted to be within the normal range, and she was prescribed polyvinyl alcohol/artificial tear drops for symptomatic relief, which resolved her symptoms. Patient 6 was treated with acetazolamide and her electrolyte levels were monitored closely until she was at the maintenance dose.

**Table 1:** Patient demographics, repair type, outcome, and complications

Age	Sex	Location	Presenting Symptoms	Repair	Post-op additional Therapy	Outcome	Complications
47	Female	Right lateral sphenoid	Acute bacterial meningitis, right CSF rhinorrhea	Alloderm and abdominal fat onlay, contralateral nasoseptal flap	Lumbar drain 72 h	Healed sphenoid on endoscopy, no recurrence × 5 years	None
52	Female	Right lateral sphenoid	Right CSF rhinorrhea	Alloderm onlay with contralateral nasoseptal flap	None	Recurrent CSF rhinorrhea at 4 weeks post-op requiring revision of nasoseptal flap	None other than recurrence
48	Female	Left lateral sphenoid	Left CSF rhinorrhea	Alloderm onlay with left middle turbinate free mucosal graft	Lumbar drain 72 h	Recurrent CSF rhinorrhea and recurrent encephalocele on 6 month MRI, lost to follow up after 1 year	None
56	Male	Left lateral sphenoid	Left CSF rhinorrhea	Alloderm inlay, abdominal fat sphenoid obliteration, ipsilateral nasoseptal flap	None	Healed sphenoid on endoscopy, no recurrence × 3 years	None
64	Female	Right lateral sphenoid	History of bacterial meningitis with bilateral profound deafness requiring cochlear implants, Right CSF rhinorrhea	Alloderm inlay, abdominal fat sphenoid obliteration, ipsilateral nasoseptal flap	None	Healed sphenoid on endoscopy, no recurrence × 3 years	Right subjective dry eye, normal Ophthamalogic exam, treated with PVA eyedrops
45	Female	Right lateral sphenoid	Acute bacterial meningitis, right clear rhinorrhea	Alloderm inlay, abdominal fat sphenoid obliteration, ipsilateral nasoseptal flap	Acetazolamide	Healed sphenoid on endoscopy, no recurrence × 3 years	None
54	Female	Right lateral sphenoid	Right CSF rhinorrhea	Alloderm inlay, abdominal fat sphenoid obliteration, ipsilateral nasoseptal flap	None	Healed sphenoid on endoscopy, no recurrence x 2 years	None

(CSF = cerebrospinal fluid, PVA = polyvinylalcohol)

## Discussion

Lateral sphenoid sinus encephaloceles are an uncommon occurrence, but can be challenging to repair given the important surrounding structures, and the far lateral location in the sphenoid. Thought for many years to be primarily a result of a persistent Sternberg’s canal, a recent study [7] showed that this persistent lateral craniopharyngeal canal is only present in a minority of patients with laterally pneumatized sphenoid sinuses on CT scan, and that persistent elevated intracranial hypertension/idiopathic intracranial hypertension (evidenced by arachnoid pits) causing erosion of the sphenoid wall is the more likely etiology of lateral sphenoid encephaloceles. Bendersky et al. [8] reported on two challenging cases of lateral sphenoid encephaloceles, both of which failed endoscopic repair with an abdominal fat onlay and required a transcranial approach with temporal lobe dissection to resolve the CSF rhinorrhea. Similarly, Arai et al. [9] reported a case of a lateral sphenoid encephalocele that was repaired using a multilayered autologous fat, cranial bone graft, and vascularized split temporalis muscle flap. Interestingly, all three patients were female.

The endoscopic transnasal approach for repair has also been used extensively. Sano et al. [10] reported the use of a transnasal endoscopic approach with image guidance to localize, resect, and repair a lateral sphenoid encephalocele using fascia lata underlay and fat onlay, with no recurrence noted. Alexander and coworkers reported on their series of 11 patients with lateral sphenoid CSF leaks [11]. They utilized an endoscopic transpterygoid approach and multilayer repair using septal bone and tissue inlay grafts (Alloderm, Duragen, or Surgisis) and an overlay tissue graft with or without a free fat graft with an additional layer of a pedicled septal flap in certain cases. There were 13 total leaks (2 patients had bilateral repairs), and they noted only one recurrence which was successfully repaired at the second surgery. Schmidt et al [12] provided an excellent review of the surgical anatomy and techniques for the two-surgeon, four-handed transpterygoid approach to lateral sphenoid encephaloceles. They noted that if the vascular structures of the pterygopalatine fossa (internal maxillary artery and its sphenopalatine artery branch) can be preserved, an ipsilateral nasoseptal flap can be utilized. If not, the contralateral flap was recommended as the ipsilateral flap will no longer be vascularized by its named arterial supply. They also presented four of their representative cases, with no recurrent CSF leak after endoscopic repair. Kirtane et al [13] presented their series of 15 lateral sphenoid CSF leaks, all of which were repaired successfully at the first attempt. Interestingly, their series included seven male subjects and eight female subjects. They utilized an endoscopic approach with a “bath plug” type autologous fat inlay such that part of the fat is intracranial and part is extracranial, with the fat graft supported by fascia. In cases where the lateral sphenoid showed multiple defects they also utilized a modified septal mucosal flap based on the sphenopalatine artery. Settecase and coworkers [14] reported on a radiographic review of 26 patients with lateral sphenoid encephaloceles and classified 15 of 26 patients as having a type 1 spontaneous lateral sphenoid cephalocele herniating into a pneumatized lateral recess of the sphenoid sinus (typically presenting with CSF leak) and the remaining 11 of 26 patients as having a type 2 spontaneous lateral sphenoid cephalocele isolated to the greater sphenoid wing without extension into the sphenoid sinus. Similar to the study by Baran˜ano et al. [7] all 26 patients were noted to have sphenoid arachnoid pits and 16 of 26 patients had an empty or partially empty sella, again supporting idiopathic intracranial hypertension as the etiology, combined with the lateral sphenoid pneumatization.

In this series of 7 patients with lateral sphenoid encephaloceles, an endoscopic transsphenoidal approach was utilized. A statistically significant benefit for successful repair was noted with utilization of a multilayered Alloderm inlay, abdominal fat onlay, and nasoseptal flap, with the nasoseptal flap contralateral in 1 of the successfully repaired patients and ipsilateral in the remaining 4 successfully repaired patients. No statistically significant benefit was noted for use of post-operative lumbar drainage. Use of a modification of the rescue flap technique for the nasoseptal flap facilitated preservation of the ipsilateral sphenopalatine artery while still allowing wide visualization of the lateral sphenoid recess. This allowed use of an ipsilateral nasoseptal flap in the last 4 patients described, providing a broader coverage of the lateral sphenoid defect given the decreased reach needed from the flap. Additionally, utilization of the Alloderm inlay combined with the autologous fat onlay appeared to increase the success rate, allowing the repair to be more “water-tight” and preventing persistent CSF leakage around the repair that might prevent healing of the dura and bony edges of the repair. The key to successful repair of these lateral sphenoid defects appears to be use of an inlay graft to seal the defect intraoperatively, and use of multiple layers to provide a durable “water-tight” closure. Consideration of post-operative adjuncts such as acetazolamide or ventriculoperitoneal shunt may aid in the reduction of CSF pressure in the idiopathic intracranial hypertension patients more likely to present with these lateral sphenoid encephaloceles, but a well-reduced encephalocele with a robust multilayer repair appears to be the mainstay of treatment. Despite the important surrounding anatomy, the endoscopic approach appears to provide a safe and effective method of repair, with only one minor complication (ipsilateral dry eye) noted. That complication was easily managed with topical artificial tears.

Several limitations are noted in this study. The relative rarity of lateral sphenoid encephaloceles and the low number of patients limits the power of the study, but a statistical advantage was found for multilayer repair with Alloderm inlay/fat onlay/nasoseptal flap nonetheless. Additionally, the single surgeon and retrospective nature of the study increase the risks of selection and recall bias.

## Conclusions

This study presents the author’s series of lateral sphenoid encephaloceles repaired using an endoscopic approach. A statistically significant advantage in success rate was noted for the use of an Alloderm inlay, abdominal fat onlay, and nasoseptal flap vs. Alloderm onlay/nasoseptal flap or free mucosal graft onlay. Given the low patient numbers overall and the low number of onlay-only patients it is difficult to draw definitive conclusions from this limited case series. Use of a modification of the rescue flap technique allowed preservation of the ipsilateral nasoseptal flap pedicle. This study highlights the utility of the endoscopic approach, and the importance of use of a multilayered repair.

## Abbreviations

CSF: cerebrospinal fluid
MRI: magnetic resonance imaging
NSF: nasoseptal flap
PVA: polyvinylalcohol
